# Navigating time equity: Balancing urgency and inclusivity in pandemic treaty negotiations

**DOI:** 10.1371/journal.pgph.0003118

**Published:** 2024-04-22

**Authors:** Sharifah Sekalala, Shajoe Lake, Sarah Hodges, Yureshya Perera

**Affiliations:** 1 School of Law, University of Warwick, Coventry, United Kingdom; 2 Department of Global Health and Social Medicine, Kings College London, London, United Kingdom; McGill University, CANADA; APHRC, KENYA; PLOS: Public Library of Science, UNITED STATES

Since the start of the COVID-19 pandemic, there have been a surge in calls for equity to protect the world from future crises. Calls have emphasized equitable distribution of vaccines, a fair access and benefit sharing (ABS) system, and health systems strengthening, all leading to negotiation of a Pandemic Treaty [1].

Unlike previous global health agreements, the finalization of this treaty was expected within two years. Recently, the Director-General of the World Health Organization (WHO) intensified the urgency, stressing that ‘time is very short’ and countries should get on board to reach an agreement by May 2024 [2]. Upon first glance, this expedited timeline may seem justifiable. Researchers estimate that almost 16 million people died at the peak of the COVID-19 pandemic between 2021–2022, reversing a 70-year trend in declining mortality rates [3]. Given the increased chances of another pandemic, there is a compelling argument for expediting the treaty-making process to facilitate faster and more equitable access to life-saving medications. In response, countries can capitalize on this momentum to swiftly adopt a much-needed global framework aimed at bolstering access to medical countermeasures and strengthening health systems to confront inevitable future pandemics [1]. Nonetheless, this rapid pace prompts concerns about what we refer to as ‘time equity’––the fair allocation of time and resources for deliberation and decision-making among all stakeholders.

## The danger of the ‘time is very short’ rhetoric

The rhetoric that ‘time is very short’ presents a significant danger, particularly for low- and middle-income countries (LMICs) advocating for vaccine equity and an equitable ABS system. Pressure to act swiftly or risk missing out on the treaty’s promise effectively warns LMICs that this is their sole opportunity, insisting they must conform to the timeline.

Historically, this notion of being time-limited has been wielded against citizens in LMICs. In the realm of climate governance, for instance, there is a growing recognition that calls for LMICs to urgently decarbonize and transition to sustainable energy sources may disproportionately impact these countries. The push for rapid decarbonization may sideline the deliberative processes of citizens from the global south, potentially undermining their voices and agency in shaping climate policies and strategies [4]. This reflects a broader anthropological argument that citizens in high income countries (HICs) exist ‘outside of time,’ with their schedules and priorities taking precedence over those in LMICs [5]. This critique also resonates with a broader understanding of how urgency may undermine deliberative processes and democratic principles, prioritizing speed over thorough consideration and inclusive participation.

Anthropologists have consistently highlighted how urgency has been historically wielded as a tool to marginalize certain groups. Western conceptions of time, for example, have often been employed to denigrate Indigenous cultures and their perspectives on time, portraying them as “primitive” or “backward” and thus justifying colonial domination [5]. Moreover, in many LMICs affected by settler colonialism, states of emergency were frequently declared, leading to overnight changes in legislation that served to legitimize the continued colonization of these countries [6]. Urgency, in this context, is not merely a call to action but a mechanism that reinforces existing power dynamics. By framing the negotiation timeline as urgent and non-negotiable, it effectively sidelines the voices of LMICs, positioning them as less relevant to the urgency of the moment and perpetuates their marginalization. This fundamental flaw in the current approach to treaty negotiations stands in stark contrast to principles of equity.

Multiple reports from Geneva suggest that HICs and LMICs alike remain intractable. Key issues dividing negotiators include the sharing of genomic data on pathogens and the creation of sustainable financing mechanisms to support treaty implementation [7]. With less than three months remaining, delegates from HICs and LMICs alike express pessimism about reaching an agreement [8, 9]. Further, many of the initial equity and accountability elements of the treaty have been diluted by a handful of rich nations, with very few enforceable obligations [10].

## Resource disadvantage

Throughout history, LMICs have encountered inherent challenges in multilateral treaty negotiations, primarily stemming from resource inequities and knowledge asymmetries. Unlike their HIC counterparts, establishing diplomatic ties and exchanging expertise around shared objectives typically demands more time for LMICs [11–13]. While HICs often boast extensive diplomatic teams with broad subject matter knowledge, many LMICs are represented by single-person delegations lacking equivalent expertise [12–14]. Even if they possess scientific proficiency, they may lack the technical legal resources required to ensure that high-level commitments are effectively translated into enforceable obligations.

## Negotiation dynamics

Negotiation dynamics further exacerbate the challenges faced by LMICs. For instance, Namibia, alongside Brazil, Indonesia, and Thailand, representing 72 LMICs, spearheaded a significant effort at the WHO. This initiative aimed to compel manufacturers to share benefits, including annual financial contributions, when utilizing genetic resources from LMICs for vaccines, therapeutics, and other health product development. However, there is growing concern about pressure on LMICs which could create shifts in negotiations [14].

Moreover, there are unfavorable dynamics in negotiation coordination. Some countries have proposed holding informal sessions to expedite negotiations due to time constraints, but resistance from certain LMICs persists, viewing this approach as undermining the process’s integrity. While some countries have formed subgroups to address specific provisions, smaller delegations prefer sequential discussions over parallel sessions, fearing that the latter may enable larger countries to engage in non-transparent talks, effectively excluding others from crucial discussions [9].

## So, what now?

The urgency surrounding the negotiation process may unfairly disadvantage LMICs, especially those with limited delegation capacity. To address this, ample time should be provided for LMICs to engage in meaningful dialogue and collective strategizing. Balancing the need for expediency with inclusivity and transparency is crucial to safeguard the interests of all parties involved.

Regarding process, the optimal approach is to view the initial May 2024 deadline as a moment for reassessment, not a final agreement. This would allow for evaluating progress and providing LMICs with additional time for collaborative efforts on key issues. Alternatively, a consensus could be reached on key issues that must be agreed upon by May 2024, with a redesigned inclusive process to effectively use the next three months.

Regarding outcomes, LMICs may choose to focus on a few key objectives. These could include identifying at least three priority issues for immediate inclusion in the treaty, while deferring other substantive matters to subsequent protocols where they retain decision-making authority. Priority areas highlighted by LMICs include time-bound waivers of intellectual property rights and an equitable access and benefit-sharing system backed by commitments for sustainable financing for health system strengthening [15]. Ancillary concerns such as providing resources for national focal points and flexibility for longer transitional periods should also be addressed.

Equity was the driving force behind countries’ participation in negotiations, with the promise of a more just response to future pandemics. However, the negotiation process to design the instrument to achieve this does not align with this vision. To design a treaty that delivers fair outcomes, attention must be given to both the process and the results. Focusing on these critical areas over the next three months will demonstrate a genuine commitment to crafting a treaty that produces equitable outcomes. Otherwise, the past year would have been a costly misuse of resources.
